# The role of germanium in diseases: exploring its important biological effects

**DOI:** 10.1186/s12967-023-04643-0

**Published:** 2023-11-08

**Authors:** Xiao Luo, Jiaxue Sun, Deshenyue Kong, Yi Lei, Fangyou Gong, Tong Zhang, Zongwen Shen, Kunhua Wang, Huayou Luo, Yu Xu

**Affiliations:** 1 Yunnan Technological Innovation Centre of Drug Addiction Medicine, Yunnan University, Kunming, 650032 China; 2 Department of Gastrointestinal and Hernia Surgery, First Affiliated Hospital of Kunming Medical University, Kunming, 650032 China; 3 Yunnan University, Kunming, 650032 China

**Keywords:** Germanium, Inflammation, Biological activity, Antioxidation, Tumor

## Abstract

With the development of organic germanium and nanotechnology, germanium serves multiple biological functions, and its potential value in biochemistry and medicine has increasingly captured the attention of researchers. In recent years, germanium has gradually gained significance as a material in the field of biomedicine and shows promising application prospects. However, there has been a limited amount of research conducted on the biological effects and mechanisms of germanium, and a systematic evaluation is still lacking. Therefore, the aim of this review is to systematically examine the application of germanium in the field of biomedicine and contribute new insights for future research on the functions and mechanisms of germanium in disease treatment. By conducting a comprehensive search on MEDLINE, EMBASE, and Web of Science databases, we systematically reviewed the relevant literature on the relationship between germanium and biomedicine. In this review, we will describe the biological activities of germanium in inflammation, immunity, and antioxidation. Furthermore, we will discuss its role in the treatment of neuroscience and oncology-related conditions. This comprehensive exploration of germanium provides a valuable foundation for the future application of this element in disease intervention, diagnosis, and prevention.

## Introduction

Germanium (Ge) is a relatively rare metal, with a chemical symbol of Ge, that belongs to the carbon group of elements and is found in nature in minerals [1]. Germanium can also be extracted and refined from ores containing high levels of germanium, typically through methods such as smelting and extraction. Chemically, germanium is stable and its atomic structure is similar with silicon (Si), as it has four outer electrons [2]. The crystal structure of germanium is face-centered cubic, with atoms connected to each other through covalent bonds, forming the germanium crystal structure [3]. This crystal structure exhibits properties that are similar to both metals and non-metals in terms of its chemical and physical characteristics. Germanium has attracted considerable attention due to its potential applications in optoelectronics, biochemistry, and medicine.

Germanium can be divided into two main forms: inorganic and organic germanium. In industry, inorganic germanium is primarily used as semiconductor materials and in optical reactions [4]. It is widely utilized in optoelectronics due to its remarkable ability to regulate and control optoelectronic properties. Additionally, germanium finds applications in high-tech fields such as catalysts, optical glasses, and infrared optical devices [5, 6]. Germanium is widely recognized as a crucial trace element, particularly essential for maintaining the normal function of the immune system and playing a key role in disease prevention [7, 8]. Conversely, a deficiency in germanium has been linked to the development of various diseases, constituting a significant factor in carcinogenesis [9]. However, due to its hydrophobic nature, inorganic germanium is rarely employed in biomedical applications. The development of water-soluble organic derivatives of germanium is intricately connected to the N. D. Zelinsky Institute of Organic Chemistry of the Russian Academy of Sciences (ZIOC RAS) and its esteemed scientists. In 1965, the first series of water-soluble derivatives were discovered by Professor S. P. Kolesnikov [10, 11]. These water-soluble compounds were synthesized through the hydrolysis reaction of the HGeCl3 adduct with cyclohexanone or methylmethacrylate. Later in 1967, Professor V. F. Mironov, a former researcher at the institute, similarly synthesized another stable water-soluble bis(carboxyethyl germanium) sesquioxide (Ge-132, CEGS), which remains the most renowned germanium compound to date [12, 13]. Since 1976, the Japanese scholar Kazuhiko Asai was the first person to recognize the drug potential of Ge-132 [14]. Organic germanium is known to possess various biological and pharmacological activities, and is often used in medicine for purposes such as anti-tumor, antiviral, antibacterial, antioxidant, immune regulation, production of hypoglycemic lipids, scavenging free radicals, and stimulating the hematopoietic system [15]. There has been increasing interest among researchers in a novel area of study concerning stable germanium analogs as essential intermediates in organic reactions. This field encompasses a diverse range of compounds, including germanium central cations, free radicals, anions, ionic free radicals, germanenes, multi-bond organogermanium compounds, germanium aromatic hydrocarbons, and donor–acceptor complexes of low coordination germanium [16]. These organogermanium compounds serve as crucial subjects of research within the realm of germanium chemistry, providing valuable insights into their chemical properties, reaction mechanisms, and potential applications.

Currently, water-soluble organic germanium compounds have found application as supplements or food additives in cosmetic products [17]. Notably, Asaigermanium demonstrates various biological activities and holds promise for diverse medicinal functions. For instance, it exhibits potential in the treatment of conditions associated with oxidative stress, autoimmune disorders, anti-tumor effects, as well as displaying antibacterial and antiviral properties [18]. In recent years, with the advancement of nanotechnology, nanostructured germanium has emerged as a promising biomedical material. Nano-germanium exhibits physical and chemical properties similar to nano-silicon, along with bio-affinity (no toxic impurities) [19] and water solubility [20]. Consequently, germanium has garnered substantial interest in various biomedical applications, including biosensors, imaging, and therapy [21, 22]. However, despite the significant potential of germanium in biology and medicine, research on its utilization remains limited, with a lack of comprehensive evaluations. Consequently, this review aims to elucidate the biological activity of germanium and explore its potential role in the treatment of cardiovascular, neuroscience, and oncology disorders.

## The biological activity of germanium

### The inhibitory effect of inflammation

Ge is a trace element necessary for nutrition and healthcare, and it is typically not stored in specific tissues or organs. Ge can be consumed through drinking water or food, or administered via injection. Germanium is absorbed by hydrochloric acid or enzymes and then transported to tissues and organs through the bloodstream in order to carry out its biological function [23]. Endotoxin, the main component of the *Escherichia coli* cell membrane, can activate the nuclear factor-κB (NF-κB) and mitogen-activating protein K pathways, leading to the excessive secretion of tumor necrosis factor-α, IL-1β, and IL-6 [24]. Animal studies have shown that Ge can inhibit inflammation by suppressing the activation of NF-κB and MAPK pathways, as well as reducing the expression of TNF-α, IL-1β, and IL-6 [8].

**Fig. 1:**
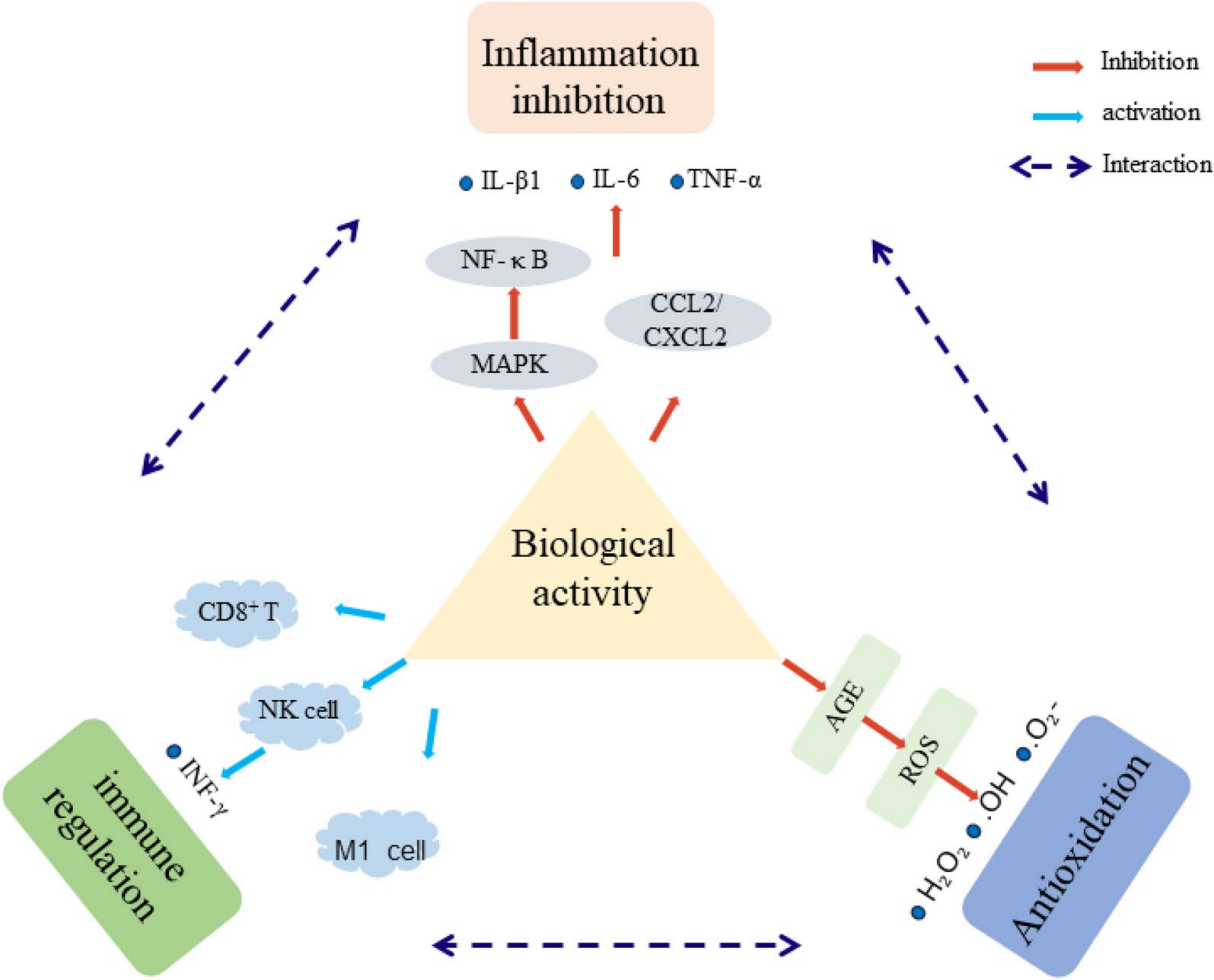
The regulatory mechanism of germanium’s biological activity

Sesquisiloxane germanate is the first germanium compound that has shown an antiviral effect. This compound plays a significant role in the development of inflammatory responses caused by viral infections [25]. Poly-trans [(2-carboxyethyl) germasesquioxane] (Ge-132), which is an organic germanium, undergoes hydrolysis to form 3-(trihydroxygermyl) propanoic acid (THGP) when dissolved in aqueous solutions. This compound has the ability to reduce inflammation through multiple mechanisms. Previous studies have shown that THGP can form a complex with a cis-diol structure and inhibit the release of IL-1β in an ATP-dependent manner [26]. THGP has the ability to inhibit the expression of IL6 and CXCL2 genes or proteins which are downstream of Reactive oxygen species (ROS) in the inflammatory pathway. This inhibition can potentially lead to reduced inflammatory signaling and subsequent cell death [17]. Furthermore, THGP exhibits a dual role in retinoic acid-inducible gene I (RIG-1)-mediated viral infection and viral replication during influenza virus infection [27]. Therefore, THGP holds great potential as a novel treatment or preventive drug for inflammatory body-related diseases. Additionally, THGP has been found to prevent sulfide-induced enhancement of Ca+ channel-dependent membrane current, consequently reducing Cav3.2-dependent pain caused by exogenous and endogenous sulfides [28]. In addition, another type of organic germanium called Spirogermanium (SG) is a nitrogen heterocyclic compound. Preliminary animal experiments have shown that Lewis rats, when administered an effective dose of SG, experience suppressed autoimmune encephalomyelitis [29]. These findings indicate that germanium can effectively combat inflammation by inhibiting inflammatory mediators and reducing the infiltration of inflammatory cells.The biological activity of germanium is shown in Fig. 1.

### The impact of immune regulation

Germanium’s immune properties, which include inducing interferon, macrophages, T suppressor cells, and enhancing natural killer cell activity, suggest that germanium may play a significant role in disease treatment [30]. It was found that significant interferon (IFN) activity was detected in the serum after oral administration of the organic germanium compound Ge-132 (300 mg/kg) in mice. Furthermore, IFN was shown to mediate mouse NK cell activity and activate macrophages [31]. Common water-soluble organic germanium compounds (Ge-132) have been found to increase immune activity. Oral administration of organic germanium Ge-132 can increase the level of α-tocopherol in the plasma and regulate liver gene expression profiles, promoting immune activation in mice [32]. The M1 macrophage is a key cell type associated with tumor immunology and is capable of engulfing cancer cells. Ge-132 and its hydrolysate THGP have been found to induce IFN-γ activity in vivo by activating NK cells and macrophages [33]. Ge-132 can also work in conjunction with lactobacillus and oligosaccharides to exert immune effects. Animal experiments have shown that mice receiving low concentrations of Ge-132 and oligosaccharides have high levels of IgA in their feces, indicating that LB/OS with low concentrations of Ge-132 can stimulate intestinal immunity [33].

Germanium enhances body immunity by activating immune activity. Low molecular weight organic germanium can also be used as an immunotherapy adjuvant to increase the immune response to influenza vaccines [34]. The interaction between viral nucleic acid and protein factors is the key process for initiating viral genome replication mediated by viral polymerase. This process activates the pattern recognition receptor (PRR)-mediated innate immune response. On one hand, THGP directly binds to the 5′-triphosphate part of viral RNA and competes with RIG-I-mediated recognition. On the other hand, THGP directly counteracts viral replication by inhibiting the interaction between virus polymerase and the RNA genome [27]. According to reports, propagermanium (3-oxygermylpropionic acid polymer) has been used as a drug to treat chronic hepatitis B, which can reduce HBV replication and lead to seroconversion [35]. Propagermanium can enhance the function of virus antigen-specific Tc cells in virus-infected mice [36]. Studies have shown that propagermanium can reduce liver injury caused by a non-specific immune response to antigen [37]. Oral administration of organic germanium Ge-132 increases alpha-tocopherol levels in plasmodesmata and modulates hepatic gene expression profiles to promote immune activation in mice [32]. These results may reveal the mechanism of propagermanium improving clinical viral hepatitis and play a crucial role in immunotherapy.

### The anti-oxidation effect

The Maillard reaction, specifically the advanced glycation end product (AGE) formation process, induces the production of reactive oxygen species (ROS) [38]. Germanium derivatives has been reported to prevent this reaction [39]. In the present study, most data supports a more direct antioxidant effect of germanium derivatives. However, the mechanism of this antioxidant effect remains unclear. Recent studies have proposed the hypothesis that germanium derivatives catalyze the decomposition of hydrogen peroxide [40]. Trace germanium can keep hydrogen peroxide at a low level, thus inhibiting/preventing oxidative stress. It has been observed that germanium is part of the active centers of some enzymes and participates in oxidation, mainly with hydrogen peroxide, without producing harmful reactive oxygen species [40]. In fact, previous results suggest that Ge-132 has the potential to act as an antioxidant supplement by protecting cells from oxidative damage [41]. Generally, antioxidants such as ascorbic acid and polyphenols are added to cultured cells at micromolar concentrations. Currently, the antioxidative activity of Ge-132 is not superior to that of other antioxidants. Thus, it is crucial for future studies to prioritize the identification of effective derivatives of Ge-132.

Germanium has numerous biological activities, which include enhancing interferon production, activating natural killer cells and macrophages, and regulating the immune system. Additionally, germanium also plays an active role in antioxidant stress. Several studies have reported the antioxidant activity of Ge-132, such as its ability to accelerate aging and induce low-density lipoprotein oxidation in spontaneous familial hypercholesterolemia model rats, porcine oocytes, and monkey liver preparations [41–44]. Furthermore, Ge-132 has been found to possess antioxidant activity in rodent bile as well [32]. Previously, it was also demonstrated that Ge-132 played a role in oxidative stress models, such as para-quart poisoning and low-density lipoprotein oxidation [43]. The Maillard reaction, particularly the formation of advanced glycation end products (AGE), leads to the production of reactive oxygen species (ROS) [45]. Ge-132 has been reported to prevent this reaction [46]. In the current research, Ge-132’s electron scavenging activity is considered to be one of the main mechanisms [47]. Ge-132 has a unique chemical structure with a Ge-C bond, allowing for electron transfer between Ge and free radicals. Hydrogen peroxide induces oxidative stress and ROS production in cultured cells. Cell experiments have also demonstrated the effectiveness of Ge-132 against oxidative stress induced by hydrogen peroxide [14]. Therefore, Ge-132 may serve as an antioxidant supplement by protecting cells from oxidative damage.

With the widespread application of 2D nanomaterials, hydrogenation has emerged as a promising method to modulate the band gap of selected nanocatalysts for therapeutic purposes [48]. Hydrogenation transforms germanium into a semiconductor with a direct band gap, referred to as hydrogen-terminated germanium (H-Germane). This H-Germane material can serve as an electron donor and exhibit antioxidant properties. Recent studies have demonstrated that H-Germanene nanoparticles possess a remarkable ability to scavenge reactive oxygen species (ROS), even at low concentrations. Furthermore, H-Germanene exhibits high biocompatibility and exerts a cytoprotective effect against oxidative stress [49].

### Germanium in anti-tumor applications

**Fig. 2:**
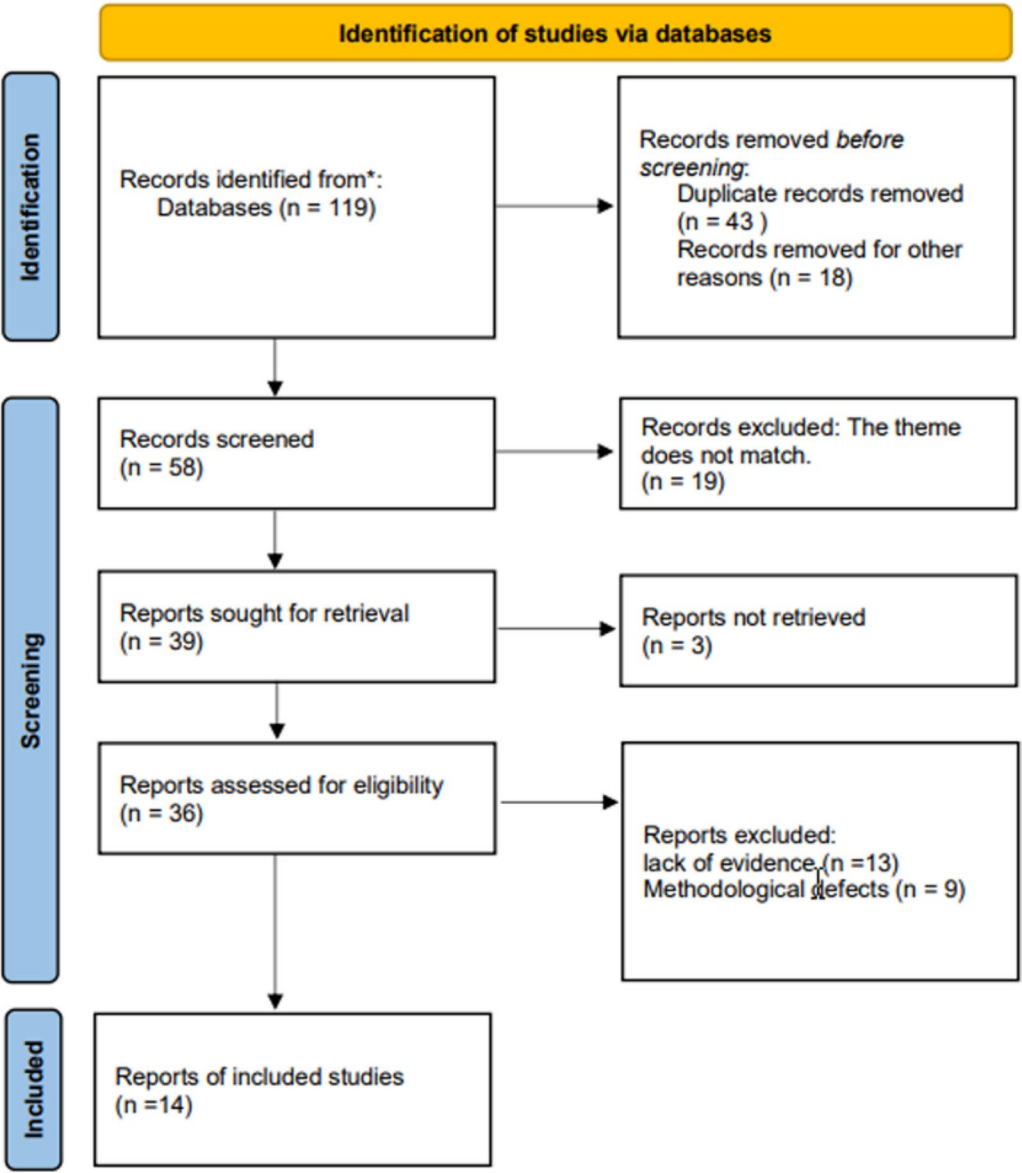
The flow chart showing the methods and strategies of the review

To further elucidate the mechanism of germanium in tumor development, we selected articles based on the recommended report items outlined in the guide for systematic review and meta-analysis (PRISMA). We conducted searches in MEDLINE, Web of Science, and Scopus databases to identify articles relevant to the association between germanium and cancer. Multiple search terms were utilized, including germanium, organic germanium, tumor, and cancer. Our inclusion criteria encompassed observational, prospective or retrospective studies, cross-sectional studies, case–control studies, cohort studies, and intervention studies. Our focus was on understanding the biological effects of germanium in disease and providing scientific evidence regarding its efficacy in treating tumor diseases. Experimental models involving humans and animals, as well as in vitro experiments, were considered. Studies lacking sufficient evidence or methodological flaws were excluded. However, it should be noted that the purpose of this review was to provide an overview of the current research landscape. We did not request additional data from the authors of published reports, nor did we analyze any data not included in the selected articles from the systematic review. The specific process is illustrated in Fig. 2.

Germanium has a wide range of applications in oncology, and numerous studies are currently investigating the low-toxicity antitumor drug activity of organogermanium compounds (See Table 1). Germanium plays a pivotal role not only in normalizing the immune system but also in facilitating the restoration of oxygen respiration (oxidative phosphorylation) in cancer cells, thereby significantly contributing to cancer prevention [50]. Germanium compounds exhibit the capacity to inhibit abnormal glycolysis, effectively neutralizing its consequences, and restoring normal biochemical parameters, oxygen respiration, and mitochondrial function in cancer cells [51]. Consequently, this comprehensive action effectively impedes, or in some cases, halts the growth of Warburg-like tumors, highlighting the potential of germanium compounds in the development of strategies for cancer prevention and treatment.

These studies have identified several types of organogermanium compounds with significant antitumor effects [52], For example, there are organogermanes sesquioxides included in this study. The organogermanes sesquioxides consist of bis-beta-carboxyethyl germanium sesquioxide (CEGS), Ge-132 (R-Ge-1), and bis-beta-carbamoylethyl germanium sesquioxide (R-Ge-2). Additionally, an organophosphorus compound with antitumor and antiviral activity, adenosine-5′-thiophosphoric acid triethylamine (5′-AMPS), was also included in the study [53]. CEGS enhances the activity of NK cells and macrophages by inducing interferon-γ (IFN-γ) activity, which exerts anti-tumor effects and inhibits tumor and metastatic growth [54]. Ge-132, functioning as an immune enhancer, exerts an anti-tumor effect by regulating immune cells and cytokines within the tumor microenvironment, including macrophages, NK cells, and IFN-γ [55]. Ge-132 has been shown to have antitumor effects in mice and rats and has been used clinically. In 1985, it was found that oral administration of Ge-132 (300 mg/kg), to mice resulted in significant interferon (IFN) activity detected in the serum, which mediates NK cell activity in mice and activates macrophages [31]. Interferon (IFN) is another drug used to treat multiple myeloma, and Propagermanim is an IFN inducer. A study found that among 10 patients with multiple myeloma treated with 10 to 40 mg of Propagermanium, two patients experienced complete remission, two patients experienced partial remission, four patients had stable disease, and two patients had progressive disease [56]. Ge-132 also has antitumor effects in C57BL/6 mice with Lewis lung carcinoma [57]. In 2000, it was discovered that oral germanium sesquioxide could be used to treat spindle cell carcinoma, resulting in rapid symptomatic remission and no clinical or imaging abnormalities observed at 42 months after starting replacement therapy [58]. Furthermore, organogermanium compounds were found to be potentially effective against certain ascites tumors through the expression of macrophages and/or T lymphocytes [59]. Ge-132 has demonstrated an anti-melanogenic effect. The organogermanium compound 3-(trihydroxygermyl) propanoic acid (THGP) was identified as a useful substrate for inhibiting melanogenesis, and its efficacy was enhanced when combined with triglyceride [60].

**Table 1:** The characteristics of germanium in cancer treatment

Study	Diseases	Types of Ge	Object	Biological effect	Conclusion
Jao et al. [68]	Intestinal cancer	(1) Organic germanium [(GeCH_2_CH_2_COOH)_2_O_3_] (2) Inorganic germanium (GeO_2_) (3) Natural organic germanium,	Sprague–Dawley male rats	Cytotoxicity	Natural organic germanium has the best preventive effect on colorectal cancer, followed by organic germanium, inorganic germanium has no anticancer effect
Guo et al. [69]	Breast cancer	Quercetin surface-functionalized germanium nanoparticles (Qu-GeNPs)	MCF-7 cells	(1) Promote apoptosis (2) Antioxidation	Qu-GeNPs has strong hydroxyl scavenging and proliferation inhibition on MCF-7 cancer cells, as well as strong apoptosis induction
Lu et al. [70]	Liver cancer	Dihydroartemisinin-organogermanium (DHA-Ge)	HepG2 cells	(1) Immune regulation (2) Antioxidation	DHA-GE has a good synergistic and anti-tumor effect and can be used as a suitable drug for tumor therapy
Kikuchi et al. [71]	Refractory cancer	Propagermanium	Human	Immune regulation	Propagermanium can induce the maturation of NK cells and may enhance its anti-tumor activity
Masuda et al. [64]	Breast cancer	Propagermanium	Human	Inhibit the function of CCL2	Propagermanium inhibits the metastasis of breast cancer by inhibiting the activities of CCL2 and IL-6
Gao et al. [72]	Liver cancer	Ge/GeO2	(1) Female Balb/c nude mice (2) HepG2 cells	(1) Antioxidation (2) Thermal energy effect	M-Ge/GeO2 is a promising system for targetable photothermal/photodynamic synergetic cancer treatment
Yumimoto et al. [62]	(1) Gastric cancer (2) Breast cancer (3) Pancreatic cancer (4) Colorectal cancer	Propagermanium	Cancer cells	(1) Inhibit inflammatory reaction (2) Inhibit the function of CCL2	Propagermanium restricts the development and metastasis of cancer by inhibiting CCL2-CCR2 pathway
Jang et al. [73]	(1) Lung cancer (2) Liver cancer (3) Thyroid cancer	Germanium	Male F344 rat	Not assessed	Germanium can significantly inhibit the development of liver nodules and lung and thyroid adenoma
Zhang et al. [52]	(1) Nasal pharyngeal cancer (2) Liver cancer (3) Colonic cancer	Ge-132	(1) Balb/c nude mice	Inhibit inflammatory reaction	Organic germanium sesquioxide has excellent anti-tumor activity, high tumor uptake and slow clearance in tumors
Azumi et al. [33]	Melanoma	3-(Trihydroxygermyl) propanoic acid (THGP)	RAW 264.7 cells and B16 4A5 cells	Immune regulation	THGP promotes M1 polarization of macrophages, and inhibits the expression of signal regulatory protein α(SIRP-α) in macrophages and CD47 in cancer
Hunakova et al. [74]	Breast cancer	(1) Tributylgermanium chloride (TBGe) (2) Triphenylgermanium chloride (TPGe)	MDA-MB-231 Cell	Not assessed	TBGe and TPGe slow down the migration of human breast cancer cells
Azumi et al. [60]	Melanoma	3-(Trihydroxygermyl) propanoic acid (THGP)	B16 4A5	Synergistic effect with kojic acid	The synergistic action of THGP and kojic acid enhanced the inhibition of melanin production
Mainwaring et al. [58]	Spindle cell carcinoma	Germanium sesquioxide	Human	Not assessed	Germanium improves the symptoms of spindle cell carcinoma
Kumano et al. [57]	Lung cancer	Ge-132	B6 mice	Not assessed	Organic germanium compound Ge-132 may have inhibitory effect on the growth and metastasis of local tumor in 3LL

Recent studies conducted in 2023 also indicated that THGP, a hydrolysis product of Ge-132, can promote M1 polarization in macrophages. Moreover, it inhibits the expression of signal-regulated protein Α in macrophages and CD47 in cancer, while suppressing the proliferation of melanoma cells through phagocytosis. These findings suggest that THGP may serve as a novel regulatory agent for tumor immunity [33]. Furthermore, THGP demonstrates inhibitory effects on melanin synthesis by mushroom tyrosinase and levodopa complex formation in B16 4A5 melanoma cells. It also acts synergistically with tretinoin as a melanogenesis inhibitor, enhancing its efficacy through binding [60]. The chemokine C-C motif chemokine 2 (CCL2) and its receptor C-C chemokine receptor type 2 (CCR2) are crucial components of the inflammation signaling axis. The CCL2-CCR2 pathway promotes cancer progression by supporting cancer cell proliferation and survival, inducing cancer cell migration and invasion, and stimulating inflammation and angiogenesis [61]. Remarkably, Propagermanium has been found to inhibit the CCL2-CCR2 signaling pathway, suggesting its potential as an important target in cancer therapeutics research [62]. Treatment of a mouse model with colon cancer using Propagermanium revealed that it reduced the number and size of tumors, attenuated adenocarcinoma changes in colon tumors, and reduced tumor-associated macrophages (TAM) [63]. A completed clinical trial evaluated the safety and efficacy of Propagermanium in 45 subjects with diabetic nephropathy who were treated with Irbesartan (NCT03627715). In human breast cancer patients, peripheral blood expression of FBXW7 was found to correlate with serum CCL2 concentrations and disease prognosis. FBXW7-deficient mice exhibited elevated serum levels of the chemokine CCL2, leading to recruitment of monocyte myeloid-derived suppressor cells and macrophages, thereby promoting metastatic tumor growth. The use of the CCL2 inhibitor Propagermanium inhibited metastasis. Inhibition of premetastatic niche formation blocked the enhancement of metastasis in FBXW7-deficient mice [63]. In a phase I dose-escalation trial in 2020, Propagermanium was evaluated for dose-limiting toxicity (DLT) as an antimetastatic agent in breast cancer patients, and its maximum tolerated dose (MTD) in patients with primary breast cancer in the perioperative period was determined to have a manageable safety profile [64].

### Germanium in neuroscience

Germanium is known to have the potential to protect nerve cells and promote their survival and repair. It may also have a neuroprotective effect on neurodegenerative diseases such as Parkinson’s and cerebral ischemia. Germanium can also influence neurotransmitters and neuromodulation by modulating neurotransmitter release and signaling, thereby exerting neuromodulatory effects. A previous study demonstrated that organic germanium monocarboxyethylgermanium sesquioxides (CGS) had a protective effect on the levels of malondialdehyde (MDA), a lipid peroxidation product, in rat hippocampal tissue after cerebral ischemia–reperfusion. CGS also significantly protected the activities of superoxide dismutase (SOD) and glutathione peroxidase, indicating an inhibitory effect on oxygen radical production and enhancement of the function of the endogenous oxygen radical scavenging system. This protective effect was observed in rats with cerebral ischemia–reperfusion injury [65]. In another study, germanium oxide (GeO2) was found to impact changes in brain cholinesterase (AchE) and monoamine neurotransmitters in mice caused by cadmium chloride (CdCl2). GeO2 inhibited the CdCl2-induced decrease in monoamine neurotransmitter levels [66]. GeO2 also exhibited the ability to scavenge oxygen free radicals, thereby counteracting lipid peroxidation and protecting brain cell membranes.

Also, in the early days, Ge-68 was used for PET (Positron Emission Tomography) imaging. Ge-68 is a radioactive isotope that undergoes radioactive decay to produce Ga-68 (gallium-68), which is commonly used as a radiotracer in PET. Ge-68, as a precursor for the production of Ga-68 tracer, is widely utilized in clinical diagnosis for early detection and monitoring of cancer, neuropsychiatric diseases, and other conditions. Apart from being a precursor, germanium compounds themselves can also be used as PET developers. For instance, certain organogermanium compounds have been studied for PET development, allowing the investigation of their biodistribution, metabolism, and pharmacodynamic properties in living organisms. In addition, germanium-based transmission measurements (GeTM) have been used in clinical settings for a considerable period. Although GeTM has limitations such as low photon flux and relatively high noise levels resulting in longer acquisition times and poorer quality of attenuation-corrected emission images, it offers a lower radiation dose compared to PET/CT [67].

## Conclusion

In summary, the hydrolyzed monomer TGHP derived from Ge-132 exhibits a diverse array of biological activities, including anti-inflammatory, antioxidative, anti-melanogenic, antiviral, immune-stimulatory, and tumor-inhibitory effects. Additionally, germanium compounds have demonstrated efficacy in alleviating pain among advanced cancer patients undergoing home care, offering a secure and dependable option for home-based treatment due to their lack of side effects. Consequently, Ge-132 and its hydrolyzed monomer TGHP display significant potential for clinical applications.

Future research efforts can focus on augmenting their biological activities through a comprehensive understanding of the identified compounds and their properties, as well as through the synthesis of novel derivatives. Furthermore, exploring germanium chemistry may unveil novel water-soluble germanium compounds and their associated properties. A profound comprehension of the relevant mechanisms can facilitate the targeted synthesis of new germanium compounds with specific biological activities, thereby expanding their scope of application in various disease domains.

## Data Availability

The authors confirm that the data supporting the findings of this study are available within the review.
